# Atorvastatin treatment does not abolish inflammatory mediated cardiovascular risk in subjects with chronic kidney disease

**DOI:** 10.1038/s41598-021-83273-2

**Published:** 2021-02-18

**Authors:** Renate M. Hoogeveen, Simone L. Verweij, Yannick Kaiser, Jeffrey Kroon, Hein J. Verberne, Liffert Vogt, Sophie J. Bernelot Moens, Erik S. G. Stroes

**Affiliations:** 1 Department of Vascular Medicine, Amsterdam University Medical Centers, University of Amsterdam, Meibergdreef 9, 1105 AZ Amsterdam, The Netherlands; 2 Department of Radiology and Nuclear Medicine, Amsterdam University Medical Centers, University of Amsterdam, Amsterdam, The Netherlands; 3 Department of Nephrology, Amsterdam University Medical Centers, University of Amsterdam, Amsterdam, The Netherlands

**Keywords:** Chronic kidney disease, Drug therapy

## Abstract

Individuals with chronic kidney disease are at an increased risk for cardiovascular disease. This risk may partially be explained by a chronic inflammatory state in these patients, reflected by increased arterial wall and cellular inflammation. Statin treatment decreases cardiovascular risk and arterial inflammation in non-CKD subjects. In patients with declining kidney function, cardiovascular benefit resulting from statin therapy is attenuated, possibly due to persisting inflammation. In the current study, we assessed the effect of statin treatment on arterial wall and cellular inflammation. Fourteen patients with chronic kidney disease stage 3 or 4, defined by an estimated Glomerular Filtration Rate between 15 and 60 mL/min/1.73 m^2^, without cardiovascular disease were included in a single center, open label study to assess the effect of atorvastatin 40 mg once daily for 12 weeks (NTR6896). At baseline and at 12 weeks of treatment, we assessed arterial wall inflammation by ^18^F-fluoro-deoxyglucose positron-emission tomography computed tomography (^18^F-FDG PET/CT) and the phenotype of circulating monocytes were assessed. Treatment with atorvastatin resulted in a 46% reduction in LDL-cholesterol, but this was not accompanied by an attenuation in arterial wall inflammation in the aorta or carotid arteries, nor with changes in chemokine receptor expression of circulating monocytes. Statin treatment does not abolish arterial wall or cellular inflammation in subjects with mild to moderate chronic kidney disease. These results imply that CKD-associated inflammatory activity is mediated by factors beyond LDL-cholesterol and specific anti-inflammatory interventions might be necessary to further dampen the inflammatory driven CV risk in these subjects.

## Introduction

Chronic kidney disease (CKD) is an important cardiovascular (CV) risk factor^1,2^. Even a modest decrease in renal function coincides with accelerated atherosclerosis and a significant increase in CV risk^1,3^. Since atherosclerosis is a lipid-driven inflammatory disease of the arterial wall, lipid lowering is the cornerstone in treatment and prevention of cardiovascular disease (CVD)^4,5^. Statin-induced LDL-cholesterol lowering reduces CV risk independent of baseline LDL-cholesterol levels in subjects with CKD^6,7^. However, the association between LDL-cholesterol and CV risk is attenuated compared to the non-CKD population^8^, resulting in a decreased beneficial impact of statin therapy as the estimated glomerular filtration rate (eGFR) declines^6^. Thus, in spite of contemporary lipid lowering regimens, a substantial residual CV risk remains in CKD patients^9^.

Previously, we demonstrated that subjects with mild to moderate CKD have increased arterial wall inflammation compared to healthy controls as assessed by ^18^F-FDG (fluordeoxyglucose) positron emission tomography/computed tomography (PET/CT)^10^. The systemic nature of this pro-inflammatory state is supported by the presence of activated circulating monocytes with increased chemokine receptor expression as well as increased transendothelial migration capacity^10^. In patients without CKD, intensive LDL-cholesterol lowering was found to reduce this inflammatory state, both at the level of the arterial wall^11,12^ and in circulating monocytes^13^. In patients with CKD, a variety of specific mediators have been suggested to contribute to inflammatory activation beyond the aforementioned lipid particles^14,15^. Whether statin treatment is capable of dampening inflammation associated with impaired renal function remains to be established.

Here, we investigated the extent of statin-mediated lowering of inflammatory activity in subjects with CKD. To this end, we assessed arterial wall inflammation and monocyte phenotype before and after 3 months of potent statin treatment in subjects with stage 3 or 4 CKD.

## Patients and methods

### Study design and population

Fourteen patients with chronic kidney disease stage 3 or 4, defined by an eGFR 15–60 mL/min/1.73 m^2^ and aged ≥ 50 years, were included in a single center, open label study with atorvastatin 40 mg once daily for 12 weeks. Exclusion criteria included current statin use, use of drugs altering cytochrome P450 3A4 metabolism, use of anti-inflammatory drugs, history of CV-events (myocardial infarction, revascularization, stroke, or peripheral arterial disease), body mass index > 30 kg/m^2^, malignancy, autoimmune disorders, clinically relevant infection (high-sensitivity C-reactive protein > 10 mg/L), diabetes mellitus, or other inflammatory conditions (NTR6896, registration date 08-Dec-2017). The ethic committee of the Academic Medical Center approved the study protocol. The study was conducted according to the Declaration of Helsinki. All study subjects provided written informed consent prior to study enrollment. Based on previous studies, a sample size of 14 subjects would have a power of 80% to yield a statistically significant difference of 8% in the most diseased segment (MDS) target-to-background ratio (TBR) (p = 0.05, 2-sided).

### ^18^F-FDG PET/CT imaging

To assess arterial wall inflammation at baseline and after 12 weeks of atorvastatin treatment, ^18^F-FDG PET/CT scans were performed on a PET/CT scanner (Biograph mCT Flow, Siemens AG, Erlangen, Germany). After a fasting period of at least 6 h, 100 MBq of ^18^F-FDG was administered intravenously. 90 min post-infusion, a low-dose, non-contrast-enhanced CT-scan (40 mAs) was performed for attenuation correction and anatomic co-registration. As described previously, arterial ^18^F-FDG uptake was evaluated in the left and right carotid artery and the ascending and descending aorta^16,17^. The carotid artery with the highest ^18^F-FDG uptake at baseline was identified as the index carotid. Target-to-background ratio (TBR) was calculated from the ratio of arterial standardized uptake value (SUV) and venous background uptake (arterial SUV/ mean background SUV)^16,17^. The venous background activity was derived from the superior vena cava (for aortic SUV correction) and ipsilateral internal jugular veins (for carotid SUV correction). The most diseased segment (MDS) was determined by calculating the mean of the maximum TBR of the three adjacent slides with the highest TBR (MDS TBR). Readers, blinded for temporal sequence, using dedicated software (Hybrid Viewer version 4.17, Hermes Medical Solutions, Stockholm, Sweden) analyzed the PET/CT images.

### Baseline measurements

After overnight fasting, patients visited the hospital for medical history recording, physical examination and blood sampling. Plasma total cholesterol, high-density lipoprotein (HDL) cholesterol and triglyceride levels were analyzed with commercially available enzymatic methods. LDL-cholesterol was calculated using the Friedewald formula^18^.

### Flow cytometry

Flow cytometry analysis was performed in whole blood. Red Blood Cells (RBCs) were lysed using RBC-lysis buffer (Affymetrix, eBioscience, San Diego, CA, USA). Next, the supernatant containing the lysed RBCs was washed and incubated with the fluorescently labeled antibodies CD14, CD16, CCR2 and CCR7. Cells were washed and samples were analyzed by flow cytometry (BD FACS Canto II; Becton Dickinson, Franklin Lakes, New Jersey). Monocytes were classified according to CD14, CD16 and HLA-DR expression. The expression of the cell surface markers was calculated as delta median fluorescence intensity (∆MFI). Data were analyzed with dedicated software (FlowJo, LLC, Ashland, OR, USA).

### Statistical analyses

Data are presented as mean ± standard deviation (SD) for normally distributed data and as median [inter-quartile range] (IQR) for skewed data. Categorical variables are expressed as absolute number and percentage. Paired samples t-tests and Wilcoxon signed rank tests were used on the change after treatment where appropriate. Two-sided p-values ≤ 0.05 were considered statistically significant. All data were analyzed with SPSS (IBM SPSS Statistics, version 25, Chicago, IL, USA).

## Results

### Study population

Fourteen subjects with stage 3–4 CKD, with a mean eGFR of 39 ± 12 and a median creatinine of 141 µmol/L [106–210] were included in this study. Subjects were aged 62 [59–69] and had a mean LDL-cholesterol of 3.7 ± 1.1 mmol/L at baseline (Table 1). Six subjects had CKD due to adult dominant polycystic kidney disease (ADPKD), three subjects had hypertensive kidney disease, two subjects had IgA nephropathy and three subjects had CKD due to unknown factors. Three participants were active smokers, seven were former smokers, and four never smoked. None of the subjects started smoking nor did any of participants ceased smoking during the study period. Three months of statin treatment significantly lowered total cholesterol (p < 0.001), LDL-cholesterol (p < 0.001) and triglycerides (p = 0.035), resulting in LDL-cholesterol values of 1.7 ± 0.6 mmol/L after treatment (Table 2).

**Table 1:** Baseline characteristics.

Baseline characteristics	N = 14
Age, years	62 [59–69]
Male	6 (42.9)
Systolic blood pressure, mmHg	129 ± 13
Diastolic blood pressure, mmHg	79 ± 11
BMI, kg/m^2^	26.3 ± 3.7
**Smoking**	
Active	3 (21.4)
Former	7 (50)
Never	4 (28.6)
Creatinine, µmol/L	141 [106–210]
eGFR, mL/min/1.73 m^2^	39 ± 12
Plasma phosphate, mmol/L	1.0 ± 0.1
Plasma calcium, mmol/L	2.4 ± 0.1
Plasma urea, mmol/L	10.6 [8.9–12.9]
Total protein in urine (portion), g/L	0.07 [0.04–0.34]
Total cholesterol, mmol/L	6.0 ± 1.6
HDL-cholesterol, mmol/L	1.5 ± 0.4
LDL-cholesterol, mmol/L	3.7 ± 1.1
Triglycerides, mmol/L	1.3 [0.9–1.9]
Leukocytes, *10^9^/L	6.2 ± 2.4
hs-CRP, mg/L	2.3 [0.9–5.5]
Glucose, mmol/L	5.7 ± 0.9

**Table 2:** Change in lipid and inflammatory parameters.

Parameter	Baseline	After treatment	p-value
Total cholesterol, mmol/L	6.0 ± 1.6	3.9 ± 1.1	< 0.0001
HDL-cholesterol, mmol/L	1.5 ± 0.4	1.6 ± 0.5	0.515
LDL-cholesterol, mmol/L	3.7 ± 1.1	1.7 ± 0.65	< 0.0001
Triglycerides, mmol/L	1.3 [0.9–1.9]	1.1 [0.6–1.8]	0.035
eGFR, mL/min/1.73m^2^	39 ± 12	38 ± 13	0.445
hs-CRP, mg/L	2.3 [0.9–5.5]	2.5 [0.7–6.5]	0.975

### Arterial wall inflammation

**Figure 1:**
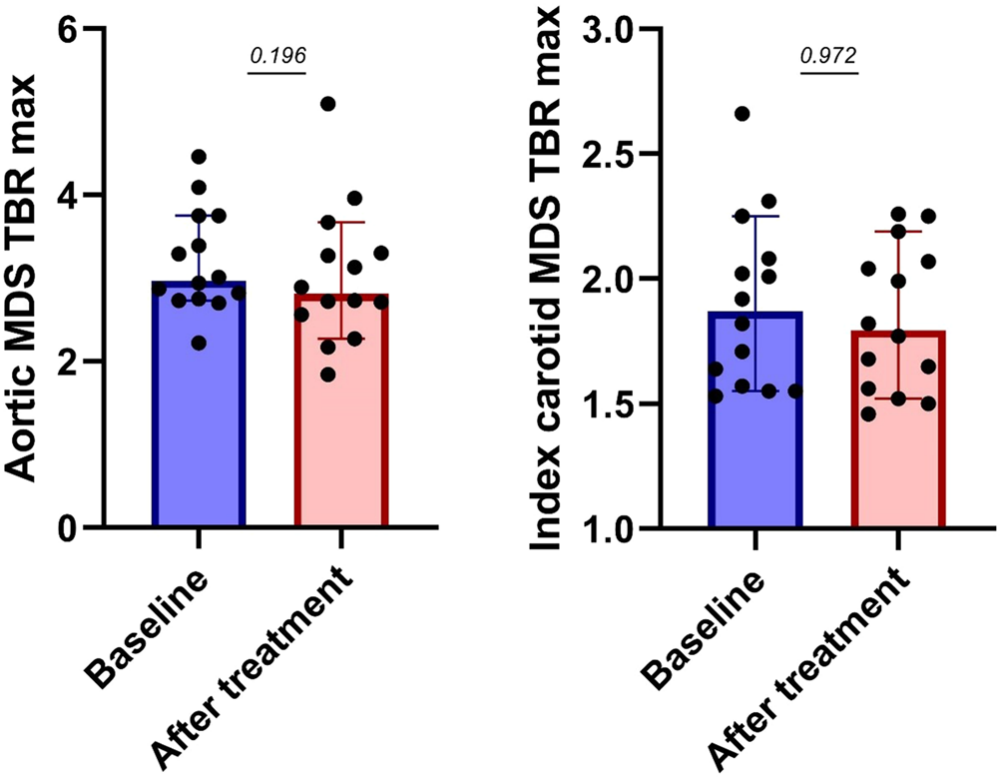
Statin treatment does not abolish arterial wall inflammation in CKD subjects. Baseline and after statin treatment arterial wall inflammation measured as mean of maximum uptake of most diseased segment target to background ratio (MDS TBR) in the aorta (left) and index carotid (right).

Median [IQR] baseline MDS TBR was 2.98 [2.75–3.75] for the aorta and 2.00 [1.74–2.25] for the index carotid. Three months of atorvastatin treatment resulted in an aortic MDS TBR of 2.89 [2.42–3.49] and an index carotid MDS TBR of 1.94 [1.77–2.42]. This change was not significant with p-values of 0.196 and 0.972 for the aortic and index carotid MDS TBR, respectively (Fig. 1). Additional PET/CT measures were directionally concordant, but also did not show significant changes after statin treatment (Table 3).

**Table 3:** PET/CT parameters.

	Baseline	After treatment	p-value
Aortic MDS TBR_max_	2.98 [2.75–3.75]	2.89 [2.42–3.49]	0.196
Aortic max TBR_max_	2.89 [2.64–3.53]	2.83 [2.36–3.47]	0.208
Index carotid MDS TBR_max_	2.00 [1.74–2.25]	1.94 [1.77–2.42]	0.972
Index carotid max TBR_max_	1.97 [1.65–2.21]	1.91 [1.72–2.25]	0.972

### Cellular inflammation

**Figure 2:**
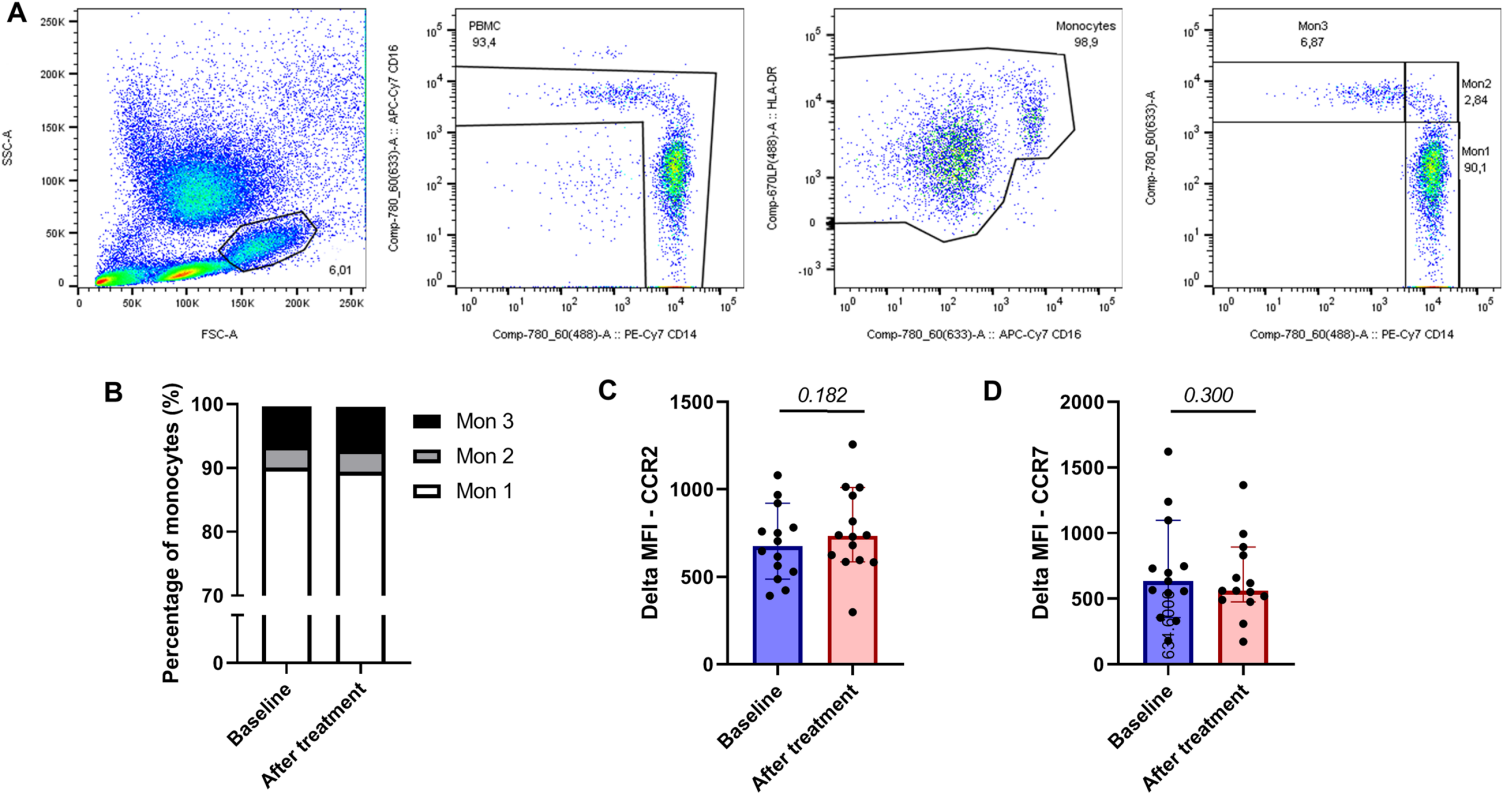
Statin treatment does not influence cellular inflammation in subjects with CKD*.* (**A**) Flow cytometry monocyte gating strategy. (**B**) Distribution across the monocyte subtypes before and after statin treatment. (**C**) Delta MFI values of CCR2 expression on monocytes before and after statin treatment. (**D**) Delta MFI values of CCR7 expression on monocytes before and after statin treatment.

Twelve weeks of atorvastatin did not change monocyte phenotype of freshly isolated monocytes. The distribution across the classical (mon1/CD14++ CD16−), intermediate (mon 2/CD14++, CD16+), and non-classical (mon 3/CD14+, CD16+) subsets did not change significantly after statin treatment (p-value 0.406, 0.694 and 0.441, respectively; Fig. 2). In line, CCR2 and CCR7 expression on monocytes (p-value 0.182 and 0.300, respectively; Fig. 2) and monocyte subsets were not affected by statin treatment.

## Discussion

This study evaluated the anti-inflammatory effects of statins on arterial wall- and cellular inflammation in subjects with stage 3–4 CKD. Three months of atorvastatin treatment resulted in a 46% reduction of LDL-cholesterol levels. Conversely, no effects were seen on MDS TBR of the aorta and carotid arteries or on chemokine receptor expression of circulating monocytes. These results imply that CKD-associated inflammatory activity is mediated by factors beyond LDL-cholesterol, and is not attenuated by statin treatment.

### Statin-refractory inflammatory state

In accordance with other subjects with increased CV-risk, subjects with mild to moderate CKD are hallmarked by a pro-inflammatory phenotype, comprising increased arterial wall inflammation and plasma immune cell activation^10,19^. In the non-CKD CV risk population, lowering of LDL-cholesterol, by either statins, LDL-cholesterol apheresis or PCSK9ab therapy, coincides with a decrease in arterial wall inflammation^11,12,20^ and in plasma immune cell activation^13^. While the arterial ^18^F-FDG-uptake in CKD subjects was comparable with CKD subjects from our earlier study, which showed significantly elevated arterial wall inflammation compared to healthy controls^10^. The absence of inflammatory improvement may imply an overriding impact of non-LDL-cholesterol factors mediating inflammatory activation in CKD. Concordantly, the pro-inflammatory phenotype of circulating monocytes in CKD-patients^10^, comprising increased CCR2 and CC7 expression, was also not affected by potent statin treatment. In analogy, we recently reported unchanged arterial wall inflammation and monocyte activation in subjects with marked lipoprotein(a) elevation following PCSK9ab therapy. In that study, the persistence of an inflammatory state could be attributed to marked residual Lp(a) elevation following the treatment^21^.

The inflammatory burden in CKD has been attributed to a complex interaction comprising immunologic, metabolic and inflammatory components in which resident macrophages and circulating monocytes are major contributors^10,14,15^. Pathophysiologic considerations propagating the pro-inflammatory state in CKD include dysregulation of calcium and phosphate, activation of the renin-angiotensin-aldosteron system, NF-ĸB pathways and inflammasome activation related to the accumulation of uremic toxins such as trimethylamine N-oxide, p-cresyl sulfate and indoxyl sulfate derivatives^22^. Indoxyl sulfate and p-cresyl sulfate are of special interest, since they are potentially modifiable and associated with both CVD and CKD-progression in the CKD-population. P-cresyl sulfate and indoxyl sulfate are solely produced by the microbiome of the large intestine ^23^ and can impair the intestinal barrier causing translocation of bacteria and bacterial products into the circulation. This change in microbiome composition, amongst others, causes an increase in bacteria that produce uremic toxins, which may in part contribute the persistence of systemic inflammation after statin therapy in CKD^24^.

### Therapeutic implications of persistent inflammation in CKD

The mechanism behind the diminished benefit of statin therapy with progressive renal insufficiency remains to be established. Worsening of kidney function coincides with an increase in both atherosclerotic as well as non-atherosclerotic CV events. Especially in advanced CKD, a large proportion of CV events can be attributed to non-atherosclerotic disease (e.g. arrhythmia, valve calcification), which is mostly unaffected by statin therapy^25^. In this respect, the persistence of a pro-inflammatory state in CKD patients, despite LDL-cholesterol lowering may provide a clue. Following the significant CV risk reduction in CVD patients following interleukin (IL)-1β monoclonal antibody treatment, it is tempting to speculate whether CKD patients need additional specific anti-inflammatory interventions in order to further reduce their considerable residual CV risk. In support, post-acute coronary syndrome patients with CKD were observed to have a larger absolute risk reductions following IL-1β antibody compared with non-CKD patients^26^. Data evaluating other anti-inflammatory moieties in CKD patients are scarce. Treatment with allopurinol decreases uric acid and ROS production, thereby decreasing NLRP3 inflammasome activation^14,27^. Long-term treatment with allopurinol was reported to attenuate renal function deterioration as well as CVD-risk in subjects with CKD^28,29^. Conversely, recent studies argue that urate-lowering treatment with allopurinol does not slow the decline in eGFR^30,31^. In line, several other interventions targeting uremic toxins have been tested, but did not provide clinical benefit^32^. Preliminary data hint towards interventions decreasing the uremic toxin load in CKD patients using pre- or probiotics^33^.

### Strengths and limitations

This study is the first study to show the effect of statin treatment on arterial wall and cellular inflammation in the CKD population. However, this study has several limitations. First, the duration of the intervention with statins was limited. Previously, ^18^F-FDG-uptake was reported to decrease within three days after apheresis as well as four weeks after initiation and intensification of statin therapy^11^. Second, smoking might influence the degree of arterial wall inflammation. However, as only three participants were active smokers, participants were their own control and none of the subjects started or ceased smoking during this study this is unlikely to have obscured a beneficial effect of statin treatment on arterial wall inflammation. Finally, the sample size of the study was limited. Since the sample size was in line with previous studies of our group showing significant reductions in plaque inflammation^12^, it is unlikely that significant changes in MDS TBR will be observed when increasing the sample size. Taking these limitations into account, the data from this study support the presence of other causes beyond LDL-cholesterol which mediate the persistent inflammatory state in CKD.

### Conclusions

Statin treatment does not decrease arterial wall inflammation, nor does it change chemokine receptor expression of circulating monocytes in subjects with mild to moderate CKD. Specific anti-inflammatory interventions might be necessary to further dampen the inflammatory driven CV-risk in these subjects.
